# Successful Anesthesia Management of Postoperative Maternal Pulmonary Edema and Uterine Hyperactivity following Open Fetal Myelomeningocele Repair

**DOI:** 10.1155/2021/6679845

**Published:** 2021-03-05

**Authors:** Denis Snegovskikh, Konstantina Svokos, Dmitri Souza, Elizabeth Renaud, Stephen R. Carr, Mark C. Kendall, Francois I. Luks

**Affiliations:** ^1^Department of Anesthesiology, Rhode Island Hospital, The Warren Alpert Medical School of Brown University, Providence, RI 02903, USA; ^2^Department of Neurosurgery, Rhode Island Hospital, Hasbro Children's Hospital, The Warren Alpert Medical School of Brown University, Providence, RI 02903, USA; ^3^Department of Anesthesiology, Ohio University, Heritage College of Osteopathic Medicine, Athens, OH 45701, USA; ^4^Department of Pediatric Surgery, Hasbro Children's Hospital, The Warren Alpert Medical School of Brown University, Providence, RI 02903, USA; ^5^Division of Maternal-Fetal Medicine, Women and Infants Hospital of Rhode Island, The Warren Alpert Medical School of Brown University, Providence, RI 02903, USA; ^6^Division of Pediatric Surgery, Hasbro Children's Hospital, The Warren Alpert Medical School of Brown University, Providence, RI 02903, USA

## Abstract

Effective tocolysis is essential after fetal myelomeningocele repair and is associated with the development of pulmonary edema. The increased uterine activity in the immediate postoperative period is commonly treated with magnesium sulfate. However, other tocolytic agents such as nitroglycerine, nifedipine, indomethacin, terbutaline, and atosiban (outside the US) have also been used to combat uterine contractility. The ideal tocolytic regimen which balances the risks and benefits of in-utero surgery has yet to be determined. In this case report, we describe a unique case of fetal myelomeningocele repair complicated by maternal pulmonary edema and increased uterine activity resistant to magnesium sulfate therapy.

## 1. Introduction

Myelomeningocele is a neural tube defect exposing neural tissue of the spinal cord and is regarded as the most serve form of spina bifida [1]. The standard treatment for myelomeningocele is surgical intervention within the first couple of days of life to limit the occurrence of infection and neurological damage. Previous literature has demonstrated that prenatal surgery for myelomeningocele repair improved neurological outcomes at 30 months [2]. As a result, this surgical technique has become increasingly popular among neonatal surgeons and is regarded as the standard of care of prenatally diagnosed myelomeningocele [3]. However, the surgical procedure is fraught with potential maternal complications such as pulmonary edema, chorioamniotic membrane separation, increase in uterine contractility, and uterine dehiscence [2]. The increased uterine activity in the immediate postoperative period is usually treated with magnesium sulfate (United States) [4]. However, there are other tocolytic agents such as nitroglycerine, nifedipine, indomethacin, terbutaline, and atosiban (outside the US) which have also been used to combat uterine contractility [5, 6]. The optimal tocolytic regimen to suppress uterine contractions following prenatal surgery remains unclear.

We present a case of prenatal repair of myelomeningocele which was complicated by postoperative pulmonary edema and increased uterine activity resistant to magnesium sulfate therapy. The authors obtained permission and written informed consent from the patient presented in this case report.

## 2. Case Description

A 33-year-old G4P3 woman at 24 weeks and 1 day of gestation presented for prenatal repair of fetal myelomeningocele. The fetal abnormality was discovered during routine prenatal ultrasound exam and confirmed with magnetic resonance imaging (MRI) scan. Past medical history was significant for postpartum preeclampsia, mild intermittent asthma, depression, and chronic hypertension. After extensive counseling with various fetus medicine specialists including the fetal surgery team on the risks and benefits of in-utero surgery, the patient decided to proceed with prenatal surgery. Physical exam and blood tests were within normal limits on the day of surgery. The patient's weight was 90.7 kg, height was 172.7 cm, and the estimated fetal weight was 0.630 kg.

**Figure 1:**
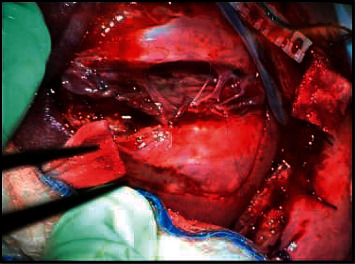
Myelomeningocele defect 5 × 5 cm associated with a kyphotic deformity and exposed neural tissue. Closure involved sewing in a skin patch over the defect.

Standard monitoring devices were used. A lumbar epidural catheter was placed uneventfully, followed by induction of general anesthesia and uncomplicated intubation of the trachea. A radial arterial catheter was then placed in the left arm. General anesthesia was maintained with sevoflurane 3.3–4% end-tidal concentration. Muscle relaxation was maintained with rocuronium. A loading dose of 6 g of magnesium sulfate (2 g prior to uterine incision and 4 g before uterine closure) followed by a continuous infusion (1 g/hr) was given to suppress uterine contractions. Hypotensive events were treated by a continuous infusion of phenylephrine at a rate of 20–40 mcg/min. Fetal anesthesia was reinforced with intramuscular injection of fentanyl 10 mcg and vecuronium 0.1 mg. The myelomeningocele defect was repaired in 69 minutes (Figure 1).

The fetal heart rate was monitored via ultrasound and was stable throughout the entire surgery. The uterine tone, which was assessed manually by an experienced Maternal-Fetal Medicine (MFM) specialist, was low during the uterine exposure. An epidural infusion consisting of bupivacaine and hydromorphone solution was initiated at the end of the operation. The patient received 900 ml of crystalloid solution, urine output was 300 ml, and the estimated blood loss was 50 ml. Muscle relaxation was reversed with sugammadex. The patient awoke from anesthesia, was extubated, and was transferred to the postanesthesia care unit (PACU). The surgical duration was 143 minutes, and no procedural complications were reported.

Upon arrival in the PACU, oxygen was given via nasal cannula and pain was well controlled with epidural analgesia. A continuous infusion of phenylephrine was initiated to minimize hypotension. The fetal heart rate and uterine contractions were monitored with cardiotocography. Thirty minutes after admission to the PACU, a significant increase in the frequency of uterine contractions required an additional dose of 2 g of intravenous magnesium sulfate. The frequency of uterine contractions did not change, and the patient complained of shortness of breath in spite of the administration of continuing oxygenation. Oxygen saturation decreased from 100% to 94%, and pulmonary auscultation revealed coarse breath sounds with rales suggesting the onset of pulmonary edema. The nasal cannula was replaced by face mask (10 L/min), magnesium sulfate was discontinued, and 20 mg of furosemide was given.

Nitroglycerine and epinephrine infusions were started (at 1 mcg/kg/min and 0.05 mcg/kg/min, respectively) with the dual goal of slowing uterine contractions and to treat the pulmonary edema. Within the next hour oxygen saturation normalized, urine output increased to 200 ml, uterine contractions became sparse, and shortness of breath disappeared. The epinephrine infusion was discontinued. The nitroglycerine infusion was slowly decreased from 0.5 mcg/kg/min to 0.3 mcg/kg/min with oxygen delivery decreased to 2 L/min. The patient was comfortable, and vital signs were stable. Uterine contractility was controlled, nitroglycerine infusion was discontinued, and the patient was discharged to the maternal unit.

The patient experienced an uncomplicated caesarean section at 36 weeks of gestation. Physical examination of the newborn revealed evidence of wound healing of the skin with the allograft patch. The newborn was able to move his lower extremities without limitations, and a computed tomography of the head revealed resolution of the cerebellar tonsillar descent and stable ventricular size. No neurological sequelae were present.

## 3. Discussion

Prenatal surgery repair is associated with chorioamniotic membrane separation, spontaneous rupture of membranes, preterm labor, placental abruption, pulmonary edema, and uterine dehiscence [7, 8]. The development of pulmonary edema following surgery can be attributed to the volume overload from the infusion and absorption of crystalloids and administration of tocolytics [9]. Previous univariate analysis demonstrated an association between prolonged tocolysis with magnesium sulfate and chorionic membrane separation and uterine scar dehiscence; although when a multivariate regression model was adjusted for clinical centers, the association was not clinically significant [10].

In our case, the patient received 900 ml of Ringer's lactate, lost 50 ml of blood, and made 300 ml of urine (1.1 ml/kg/hour during the last two hours of surgery). In addition, the epidural analgesia was initiated at the end of the surgery. It is unlikely that the volume overload was the cause of the maternal pulmonary edema. The patient received a total of 8 g of magnesium sulfate throughout the perioperative period, which was intended to provide effective tocolysis. However, it did not suppress the progression of uterine activity and was likely the contributing factor in the development of pulmonary edema in this patient. Under these circumstances, further administration of magnesium sulfate was not warranted. While loop diuretics are part of the standard therapy for pulmonary edema, the clinical effect is delayed in comparison to vasodilators and positive inotropic agents. Moreover, loop diuretics lack tocolytic properties.

There are no previous reports describing the combined administration of nitroglycerine and epinephrine infusions as dual treatment for resolving pulmonary edema and magnesium sulfate-resistant uterine contractions. It is possible that the beta-mimetic effect of epinephrine was synergetic to tocolytic effect of nitroglycerine. The dual therapy quickly resolved pulmonary edema and suppressed uterine activity.

Previous studies have investigated other agents as an alternative to magnesium sulfate [11, 12]. A prospective nonrandomized cohort study examining the tocolytic effect of atosiban after open fetal myelomeningocele repair demonstrated similar short-term uterine outcome without serious maternal complications as compared to magnesium sulfate [13]. The tocolytic agent, atosiban however, is not available in North America. A systematic review of nitric oxide donors compared with other tocolytic drugs showed no significant evidence that nitric oxide donors performed better than magnesium sulfate, calcium channel blockers, betamimetics, or a combination of tocolytics for pregnancy continuation [14]. Although there was no significant difference between groups for infant morbidity or mortality outcomes, nitric oxide donors did demonstrate less adverse events except for headaches.

Our case highlights that a combination of nitroglycerine and epinephrine infusions can be used as multimodal therapy for patients who have developed pulmonary edema and magnesium sulfate uterine resistance during the postoperative period after prenatal repair of myelomeningocele. Prospective randomized controlled trials investigating the effectiveness of combined nitroglycerine and epinephrine infusion for postoperative tocolysis and its potential role in reducing maternal complications are warranted.

## Data Availability

No data were used to support this study.

## References

[1] Juranek J., Salman M. S. (2010). Anomalous development of brain structure and function in spina bifida myelomeningocele. *Developmental Disabilities Research Reviews*.

[2] Adzick N. S., Thom E. A., Spong C. Y. (2011). A randomized trial of prenatal versus postnatal repair of myelomeningocele. *New England Journal of Medicine*.

[3] Moldenhauer J. S., Adzick N. S. (2017). Fetal surgery for myelomeningocele: after the management of myelomeningocele study (MOMS). *Seminars in Fetal and Neonatal Medicine*.

[4] Herroeder S., Schönherr M. E., De Hert S. G., Hollmann M. W., Warner D. S. (2011). Magnesium-essentials for anesthesiologists. *Anesthesiology*.

[5] Younger J. D., Reitman E., Gallos G. (2017). Tocolysis: present and future treatment options. *Seminars in Perinatology*.

[6] De Silva D. A., Sawchuck D., Dadelszen P. V. (2015). Magnesium sulphate for eclampsia and fetal neuroprotection: a comparative analysis of protocols across canadian tertiary perinatal centres. *Journal of Obstetrics and Gynaecology Canada*.

[7] Heuer G. G., Moldenhauer J. S., Scott Adzick N. (2017). Prenatal surgery for myelomeningocele: review of the literature and future directions. *Child’s Nervous System*.

[8] Moldenhauer J. S., Soni S., Rintoul N. E. (2015). Fetal myelomeningocele repair: the post-MOMS experience at the children’s hospital of philadelphia. *Fetal Diagnosis and Therapy*.

[9] Saracoglu K. T., Schuerg R., Kafali H. (2016). Advanced hemodynamic monitoring during fetal surgery. *Trends in Anaesthesia and Critical Care*.

[10] Johnson M. P., Bennett K. A., Rand L. (2016). MOMS: obstetrical outcomes and risk factors for obstetrical complications following prenatal surgery. *American Journal of Obstetrics & Gynecology*.

[11] Hoshijima H., Denawa Y., Mihara T. (2017). Efficacy of prophylactic doses of intravenous nitroglycerin in preventing myocardial ischemia under general anesthesia: a systematic review and meta-analysis with trial sequential analysis. *Journal of Clinical Anesthesia*.

[12] Klam S. L., Leduc L. (2004). Management options for preterm labour in Canada. *Journal of Obstetrics and Gynaecology Canada*.

[13] Ochsenbein-Kölble N., Krähenmann F., Hüsler M. (2018). Tocolysis for in utero surgery: atosiban performs distinctly better than magnesium sulfate. *Fetal Diagnosis and Therapy*.

[14] Duckitt K., Thornton S., O’Donovan O. P., Dowswell T. (2014). Nitric oxide donors for treating preterm labour. *Cochrane Database System Reviews*.

